# Novel S2 subunit-specific antibody with broad neutralizing activity against SARS-CoV-2 variants of concern

**DOI:** 10.3389/fimmu.2023.1307693

**Published:** 2023-12-08

**Authors:** Chang-Kyu Heo, Won-Hee Lim, Jihyun Yang, Sumin Son, Sang Jick Kim, Doo-Jin Kim, Haryoung Poo, Eun-Wie Cho

**Affiliations:** ^1^ Rare Disease Research Center, Korea Research Institute of Bioscience and Biotechnology (KRIBB), Daejeon, Republic of Korea; ^2^ Department of Functional Genomics, KRIBB School of Bioscience, University of Science and Technology, Daejeon, Republic of Korea; ^3^ Infectious Disease Research Center, Korea Research Institute of Bioscience and Biotechnology (KRIBB), Daejeon, Republic of Korea; ^4^ Synthetic Biology and Bioengineering Research Center, Korea Research Institute of Bioscience and Biotechnology (KRIBB), Daejeon, Republic of Korea; ^5^ Department of Biomedical Science and Engineering, Konkuk University, Seoul, Republic of Korea

**Keywords:** SARS-CoV-2, inactivated virus, S2 subunit, broadly neutralizing antibody, antibody dependent cellular phagocytosis

## Abstract

**Introduction:**

Severe acute respiratory syndrome coronavirus 2 (SARS-CoV-2), the causative agent of coronavirus disease 2019 (COVID-19), had a major impact on both the global health and economy. Numerous virus-neutralizing antibodies were developed against the S1 subunit of SARS-CoV-2 spike (S) protein to block viral binding to host cells and were authorized for control of the COVID-19 pandemic. However, frequent mutations in the S1 subunit of SARS-CoV-2 enabled the emergence of immune evasive variants. To address these challenges, broadly neutralizing antibodies targeting the relatively conserved S2 subunit and its epitopes have been investigated as antibody therapeutics and universal vaccines.

**Methods:**

We initiated this study by immunizing BALB/c mice with β-propiolactone-inactivated SARS-CoV-2 (IAV) to generate B-cell hybridomas. These hybridomas were subsequently screened using HEK293T cells expressing the S2-ECD domain. Hybridomas that produced anti-S2 antibodies were selected, and we conducted a comprehensive evaluation of the potential of these anti-S2 antibodies as antiviral agents and versatile tools for research and diagnostics.

**Results:**

In this study, we present a novel S2-specific antibody, 4A5, isolated from BALB/c mice immunized with inactivated SARS-CoV-2. 4A5 exhibited specific affinity to SARS-CoV-2 S2 subunits compared with those of other β-CoVs. 4A5 bound to epitope segment F1109–V1133 between the heptad-repeat1 (HR1) and the stem-helix (SH) region. The 4A5 epitope is highly conserved in SARS-CoV-2 variants, with a significant conformational feature in both pre- and postfusion S proteins. Notably, 4A5 exhibited broad neutralizing activity against variants and triggered Fc-enhanced antibody-dependent cellular phagocytosis.

**Discussion:**

These findings offer a promising avenue for novel antibody therapeutics and insights for next-generation vaccine design. The identification of 4A5, with its unique binding properties and broad neutralizing capacity, offers a potential solution to the challenge posed by SARS-CoV-2 variants and highlights the importance of targeting the conserved S2 subunit in combating the COVID-19.

## Introduction

Coronavirus disease 2019 (COVID-19) is caused by severe acute respiratory syndrome coronavirus 2 (SARS-CoV-2), a betacoronavirus (β-CoV) that was first identified in 2019 and continues to spread. As of 6 September, 2023, approximately 695 million cases and 6.9 million deaths have been confirmed (1), showing an exponentially increasing morbidity and mortality compared with SARS-CoV despite having a lower fatality rate.

In response to COVID-19, vaccine development progressed rapidly, and monoclonal antibodies were isolated from convalescent patients and used as therapeutic antibodies. However, the emergence of SARS-CoV-2 variants that evade antibody-mediated immunity due to transmission bottlenecks and immunological pressures has become a significant concern (2). An urgent need remains for the development of universal vaccines and broadly neutralizing therapeutic antibodies to address these variants of concerns.

Coronavirus infection is initiated by the spike protein (S) located on the surface of the virion (3). It binds primarily to the human ACE2 receptor, which is often the first step in a SARS-CoV-2 attack on human cells. At the molecular level, the S protein comprises 1273 amino acid (aa) residues and is composed of two subunits, namely S1 (14–685 aa) and S2 (686–1273 aa), and each S protein is composed of three S1/S2 subunits, forming a trimer. S protein trimers form a bulbous, crown-like halo surrounding the viral particle. Based on the structure of coronavirus S protein monomers, the S1 and S2 subunits form the bulbous head and stalk region (3). In the S1 subunit, there is an N-terminal domain (NTD, 14–305 aa) and a receptor-binding domain (RBD, 319–541 aa). The RBD of the S1 subunit is responsible for binding to ACE2 (4). RBD switches between a “closed” or “down” receptor-inaccessible conformation, and an “open” or “up” conformation that allows binding to the ACE2 receptor (5, 6). The receptor binding and proteolysis of spike of SARS-CoV-2 release its S2 subunit to rearrange and catalyze viral-cell fusion (7). The S2 subunit, composed successively of a fusion peptide (FP, 788–806 aa), heptapeptide repeat sequence 1 (HR1, 912–984 aa), HR2 (1163–1213 aa), transmembrane domain (TM, 1213–1237 aa), and cytoplasm domain (CT, 1238–1273 aa), is responsible for viral fusion and entry (3).

These two subunits, S1 and S2, which play important roles in viral infection, are the main targets of neutralizing antibodies for the treatment of SARS-CoV-2 (8), and neutralizing antibodies against them can reduce the severity of the infection (9). The RBD region of the S1 protein is the primary target of neutralizing antibodies. By targeting RBD, neutralizing antibodies can block its interaction with ACE2, preventing the virus from entering the cell and thus halting the infection process. They are also highly immunogenic and can easily trigger an immune response. Anti-RBD immune IgG has been considered a predictor of disease severity and survival (10). In addition to RBD, there are also antibodies against the NTD region of S1 subunit, which bind to the largest moiety surface of the NTD, mainly away from the viral membrane, and inhibit viral binding to cells (11). Except for RBD and NTD, there are also some neutralizing antibodies that bind to other regions of the S protein, including the C-terminal domain and S2 (12, 13); however, the effectiveness of these antibodies has been assessed to be less potent and less immunogenic than antibodies against S1 (8). However, during the pandemic, various mutations in the RBD region were found in several variant strains, which may reduce the neutralizing capacity of existing therapeutic antibodies and vaccine efficacy (14). Therefore, there is still a need to find more conserved neutralizing epitopes in SARS-CoV-2, and antibodies against the S2 subunit that retain a conserved sequence are still worth exploring (8, 15).

Several anti-S2 antibodies isolated from patients with COVID-19 showed neutralizing potency against SARS-CoV-2, SARS-CoV and Middle East respiratory syndrome-coronavirus (MERS-CoV) (8, 16). Two regions of S2, the FP and the stem-helix (SH, 1140-1161aa) region immediately upstream of HR2 appear to account for nearly all SARS-CoV-2 S2-specific antibodies induced by natural infection or vaccination. The antibodies targeting regions other than FP or SH were also reported, including that of HR1 or the connector domain (CD) for SARS-CoV and MERS-CoV, respectively (8, 16).

Herein, we isolated and characterized a novel anti-S2 monoclonal antibody (mAb) using a mouse hybridoma from BALB/c mice immunized with inactivated SARS-CoV-2. The anti-S2 antibody (4A5), demonstrated high affinity for S variant antigens and exhibited a distinct reactivity compared with other SH-domain reactive anti-S2 antibodies. We characterized the binding properties of 4A5 against spike protein variants and used recombinant proteins to determine its epitope. Furthermore, we evaluated its neutralizing potency against viral variants and investigated its potential to block viral infection through functions mediated by the constant (Fc) region of the antibody, including antibody-dependent cellular cytotoxicity and phagocytosis (ADCC and ADCP, respectively).

## Materials and methods

### Cell cultures

HEK293T, Raji and RAW 264.7 cells were procured from the American Type Culture Collection (ATCC, Manassas, VA, USA). HEK293T/hACE2 cell was obtained from Creative Diagnostics (Shirley, NY, USA). Cells were cultured in DMEM or RPMI1640 supplemented with 10% heat-inactivated fetal bovine serum (FBS) and antibiotics-antimycotics at 37°C in a 5% CO_2_ incubator. HEK-Blue™ hACE2-TMPRSS2, 293-hMyD88, Jurkat-Lucia™ NFAT-CD16 and Jurkat-Lucia™ NFAT-CD32 cells were purchased from InvivoGen (San Diego, CA, USA) and were cultured according to the suppliers’ protocol. HEK293T cells were transfected with SARS-CoV-2 spike (Wuhan)-His/pCMV3 vector (Sino Biological, Beijing, China) by using polyethylenimine (PEI; Polysciences, Warrington, PA, USA).

### Antibodies and antigens

For human IgG1 expression of S2P6 (17), variable region sequences of heavy and light chains were synthesized (Integrated DNA Technologies, Coralville, IA, USA) and cloned into pCEP-WPRE-hCH (hIgG1) and pCEP-WPRE-hCK for heavy and light chains, respectively, via the NEBuilder HiFi DNA Assembly system (New England Biolabs, Ipswich, MA, USA). Heavy and light chain constructs were cotransfected into ExpiCHO cells (Thermo Fisher Scientific, Waltham, MA, USA), and recombinant IgG molecules were produced and purified as previously described (18). The following antibodies were used: anti-S2 mAbs NBP2 (Novus Biologicals, Centennial, CO, USA, Cat#NBP2-90999) and MA5 (Thermo Fisher Scientific, Cat#MA5-35946), anti-RBD mAb MM43 (Sino Biological, Cat# 40591-MM43) and anti-SARS-CoV-2 NP mAb 1G6 (19). Mouse anti-His Ab was obtained from Qiagen (Hilden, Germany, Cat#34650), and rabbit anti-His Ab from Santa Cruz Biotechnology (H-15; Dallas, TX, USA, Cat#SC-803).

Antigens obtained from Sino Biological included SARS-CoV-2 antigens S1 (Cat#40591-V08H), S2 (Cat#40590-V08B), RBD (Cat#40592-V08H), nucleocapsid (Cat#40588-V08B), spike (D614G)-ectodomain (ECD; Cat#40589-V08B4), Alpha B.1.1.7 spike-ECD (Cat#40589-V08B6), Gamma P.1 spike-ECD (Cat#48589-V08B10), Delta B.1.617.2 spike-ECD (Cat#40589-V8B16), Omicron B.1.1.529 spike-ECD (Cat#40589-V08B33) and Omicron B.1.1.529 S-trimer-ECD (Cat#40589-V08H26), SARS-CoV spike-ECD (Cat#40634-V08B), MERS-CoV spike-ECD (Cat#40069-V08B), HCoV-OC43 spike-ECD (Cat#40607-V08B) and HCoV-HKU1 spike-ECD (Cat#40606-V08B). SARS-CoV-2 (Wuhan) S-trimer-ECD was purchased from Acro biosystems (Newark, DE, USA, Cat#SPN-C52H9).

SARS-CoV-2 S2-truncated mutants were constructed as shown in **Figure S1**. Gene fragments containing the serially truncated S2 mutant were amplified from SARS-CoV-2 spike (Wuhan)-His/pCMV3 vector (Sino Biologicals) by PCR and inserted into pET28a(+) (Merck Millipore, Burlington, MA, USA) via NdeI and XhoI. The constructs were transformed into SHuffle^®^ T7 *E coli* (New England Biolabs, Cat#C3026J). Recombinant protein expression was induced with 1 mM isopropyl β-D-1-thiogalactopyranoside (IPTG). Cell lysates were prepared in PBS with 1 mM EDTA and 1 mM PMSF for analysis.

### SARS-CoV-2 pseudovirus

Pseudoviruses bearing SARS-CoV-2 S variants were prepared using pLV-Spike vectors (InvivoGen) for Wuhan (prototype), D614G, Omicron BA.1 and Omicron BA.2. Lenti-X 293T cells (Takara Bio, San Jose, CA, USA) were used to produce pseudoviruses. Cells were cotransfected with pLV-Spike, pCDH-EF1a-eFFly-eGFP (Addgene, Watertown, MA, USA) and psPAX2 (Addgene) vectors using TransIT-VirusGEN transfection reagent (Mirus, Madison, WI, USA). Pseudoviruses were harvested from filtered cell culture media. Viral titer was determined via a Lenti-X™ p24 Rapid Titer Kit (Takara Bio). The infectivity of pseudoviruses were measured by quantification of the luciferase activity. Serially diluted pseudoviruses were mixed with HEK-Blue™ hACE2-TMPRSS2 or HEK293T/hACE2 cells (3 × 10^4^) in phenol red-free DMEM (100 μL) in each well of a 96-well plate. After 24 h of incubation at 37°C in a CO_2_ incubator, cells were lysed and luciferase activity was measured and relative light units (RLU) were determined using the One-Glo^®^ Luciferase Assay System (Promega, Madison, WI, USA) and GloMax Navigator Microplate Luminometer (Promega). Pseudoviruses were aliquoted and stored at -80°C.

### Mouse immunization

Five-week-old female BALB/c mice from Orient Bio (Seongnam, Republic of Korea) were acclimated for a week in a pathogen-free facility. Mice (n = 3) were immunized in the footpad three times at two-week intervals with inactivated SARS-CoV-2, S2, or S-trimer (S_trimer_) antigen. Inactivated SARS-CoV-2 was prepared by Vero cell propagation followed by β-propiolactone inactivation (20). Antigen/adjuvant mixes consisted of 10 µg antigen in 25 µL PBS and 25 µL TiterMax Gold adjuvant (Sigma-Aldrich, St. Louis, MO, USA, Cat#T2684). Serum was collected two weeks post-boost for antibody analysis.

### Selection of B-cell hybridoma producing anti-S2 monoclonal antibody

Two weeks after the final immunization of inactivated SARS-CoV-2, lymphocytes were isolated from popliteal lymph nodes and then fused with myeloma FO cells (ATCC, Cat#CRL-1646) to generate B-cell hybridoma clones (19). Monoclonal anti-SARS-CoV-2 S2 antibody-secreting B-cell hybridomas were selected via anti-S2 ELISA as detailed below. Antibodies were purified from hybridoma cell culture media with protein G agarose (Cytiva, MA, USA, Cat#17040501). The mAb isotype was determined with a Rapid Antibody Isotyping Kit (Thermo Fisher Scientific, Cat# 26178).

### Indirect ELISA

Antigens were diluted in Tris-buffered saline (TBS, pH 7.4) and coated onto Maxisorp 96-well plates (Thermo Fisher Scientific) at 100 ng/well. After blocking with 5% skim milk in TBST (TBS with Tween-20), primary antibodies were added and incubated for 2 h at 37°C. Horseradish peroxidase (HRP)-conjugated anti-mouse IgG antibody (Cell Signaling Technology, Danvers, MA, USA) or anti-human polyvalent immunoglobulins (G,A,M)-peroxidase antibody (Sigma-Aldrich) was used as the secondary antibodies. The antigen-antibody reaction was visualized with Ultra-TMB solution (Thermo Fisher Scientific). After 10 min, 0.2 M H_2_SO_4_ was added to stop color development. The color reaction was measured at 450 nm (OD_450_) with a VersaMax microplate reader (Molecular Devices, San Jose, CA, USA).

### Competitive ELISA

HRP-conjugated 4A5 was prepared using a Lightning-Link^®^ HRP conjugation kit (Abcam, Cambridge, UK, Cat#ab102890). Maxisorp 96-well plates were coated with 20 ng per well SARS-CoV-2 S2 antigen and then blocked with 5% skim milk in TBST. A mix of 10 ng HRP-conjugated 4A5 and 1 µL per well competitor mouse serum (serially diluted) was added and incubated at 37°C for 2 h. The reaction was developed using Ultra-TMB Solution.

### Western blot analysis and immunoprecipitation

Cell lysates in RIPA buffer (PBS, pH 7.4, with 1.0% NP-40, 0.1% SDS, 0.5% sodium deoxycholate, protease inhibitor cocktail), recombinant proteins or *E. coli* lysates of SARS-CoV-2 S2-truncated mutants were resolved with 12% SDS-PAGE and transferred to PVDF membrane (Millipore). After blocking with 5% skim milk in TBST, membranes were probed with anti-S2 antibodies or mouse anti-6×His antibody (1:2500; Qiagen). Antibody-reactive bands were detected via HRP-conjugated secondary reagents and chemiluminescence with ECL reagent (Cytiva). For immunoprecipitation, 800 µg lysates were mixed with 4A5 or control IgG2a at 4°C for 16 h. Protein G resin (Thermo Fisher Scientific) was used to capture antigen-antibody complexes for subsequent western blot analysis.

### Immunofluorescence

HEK293T cells transfected with SARS-CoV-2 spike-His/pCMV3 vector (expressing S: Wuhan or D614G) were cultured on coverslips and fixed with 2% paraformaldehyde in PBS for 20 min at room temperature (RT). Following blocking with 1% BSA in PBS, cells were incubated with 5 µg/mL 4A5 at RT for 1 h. After washing, Cy5-conjugated anti-mouse IgG (Abcam, Cat#ab6563) was applied for 1 h at RT. The coverslips were mounted onto microscope slides with a Vectashield solution containing 4’,6-diamidino-2-phenylindole (DAPI; Vector Laboratories, Newark, CA, USA). Stained cells were observed via confocal microscopy (Zeiss, Oberkochen, Baden-Württemberg, Germany).

### Surface plasmon resonance assay

BIAcore T200 surface plasmon resonance (SPR; Cytiva) was used to assess anti-S2 antibody affinities for the S antigen. SARS-CoV-2 spike (D614G)-ECD and SARS-CoV spike-ECD were immobilized on CM5 sensor chips by EDC/NHS activation. Antibodies were tested at six dilutions (12.5–400 nM or 6.25–100 nM) in HBS-EP+ buffer (0.01 M HEPES pH 7.4, 0.15 M NaCl, 3 mM EDTA and 0.05% Surfactant P20). Measurements were made at 30 μL/min, 25°C, in HBS-EP+ buffer. Antibody affinity was analyzed with BIAcore T200 Evaluation software 3.1 using a 1:1 (Langmuir) binding model.

### Inhibition of SARS-CoV-2 S protein binding to ACE2 via anti-S antibodies

SARS-CoV-2 S-His (1 µg) was mixed with rabbit anti-His Ab (2 µg) in 1 mL PBS for 30 min at 37°C. Serially diluted anti-spike antibodies were added and incubated for 30 min at 37°C. The mixture of S-His: anti-His Ab: anti-spike Ab was added to 4 × 10^5^ HEK293T/hACE2 cells in PBS with 2% FBS and 0.09% azide, and incubated for 40 min on ice. After buffer washing, FITC-conjugated anti-rabbit IgG (Abcam, Cat#ab6717) was applied. Quantitative FITC-stained cells were assessed via flow cytometry with BD FACSCalibur™ (BD Biosciences, Bergen County, NJ, USA).

### Pseudovirus neutralization assay

Pseudovirus, adjusted to ≥10^4^ relative light unit (RLU), was mixed with antibodies in phenol red-free DMEM (100 μL). After incubation for 1 h at 37°C, pseudovirus-antibody mixtures were added to HEK-Blue™ hACE2-TMPRSS2 or HEK293T/hACE2 target cells (3 × 10^4^) and then incubated for 24 h at 37°C in a CO_2_ incubator. Subsequently, cells were lysed and luciferase activity was measured.

### Syncytium formation assay

The inhibitory effect of anti-S2 antibodies against SARS-CoV-2 S protein-induced fusion was measured using donor cells expressing S protein and active MyD88, alongside acceptor cells with the NF-kB-SEAP gene. Donor cells were prepared by transfecting SARS-CoV-2 S protein (D614G)/pUNO1 vector (InvivoGen) into 293-hMyD88 cells using PEI. After 2 days, 1 × 10^5^ transfectants were mixed with 3-fold serially diluted antibodies (20 µg/mL starting concentration) in 96-well plates. HEK Blue™ hACE2-TMPRSS2 cells (2 × 10^4^) were added per well and incubated for 24 h at 37°C in a CO_2_ incubator. Culture supernatants were treated with QUANTI-Blue™ (InvivoGen), then incubated at 37°C for 3 h. Absorbance was measured at 620 nm.

### ADCC and ADCP assays

ADCC and ADCP were performed using Jurkat-Lucia™ NFAT-CD16 and Jurkat-Lucia™ NFAT-CD32 cells. Target cells were prepared by transfecting SARS-CoV-2 S (D614G)/pUNO1 vector into HEK293T cells with PEI. A total of 4 × 10^5^ transfectants were mixed with 2-fold serially diluted antibodies (20 μg/mL starting condition) in a 96-well plate. Effector cells, Jurkat-Lucia™ NFAT-CD16 or Jurkat-Lucia™ NFAT-CD32 (2 × 10^5^ cells), were added to wells with target cells and antibodies. The mixture was then incubated at 37°C, 5% CO_2_ for 6 h. Then, 40 μL of the coculture supernatant was mixed with 80 μL of QUANTI-Luc™ 4 Lucia/Gaussia solution (InvivoGen) per well of a 96-well white plate and luciferase activity was measured.

### Antibody-dependent enhancement assay

ADE assays were performed using RAW 264.7 and Raji cells. A total of 50 µL of serially diluted antibodies were mixed with 50 µL SARS-CoV-2 S (D614G) pseudovirus (adjusted RLU ≥ 10^4^). The mixture was incubated at 37°C, 5% CO_2_ for 1 h. Then, 2 × 10^5^ RAW 264.7 or Raji cells per well were added to the mixtures with pseudoviruses and antibodies and incubated for 24 h. Afterward, cells were lysed, and luciferase activity was measured.

### Statistical analysis

Statistical analysis was conducted using GraphPad Prism 10.0.0 (GraphPad Software). Values are presented as mean ± SD.

## Results

### An anti-S2 monoclonal antibody derived from mice immunized with inactivated SARS-CoV-2

**Figure 1:**
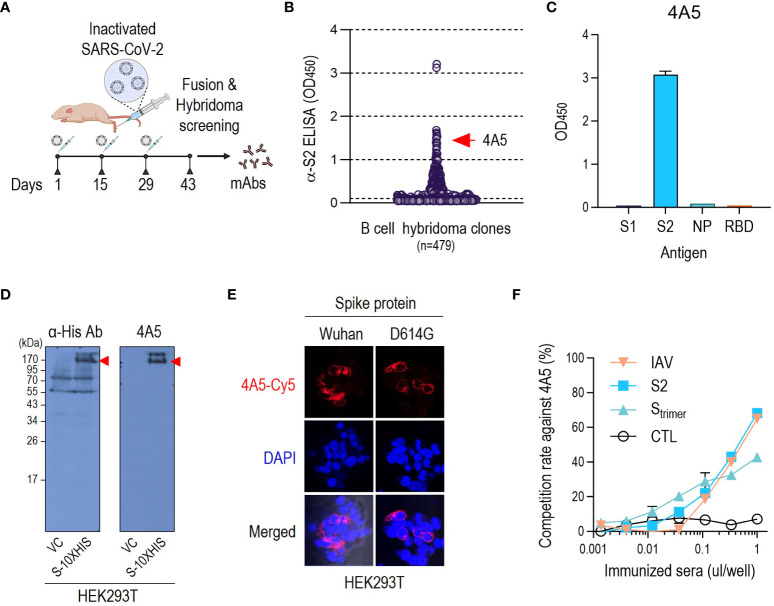
Isolation of a mouse mAb against SARS-CoV-2 S2 subunit. **(A)** Immunization strategy for discovering anti-S2 antibodies. The figure was created with BioRender.com. **(B)** ELISA screening of hybridoma culture supernatants from clones that produce antibodies against SARS-CoV-2 S2. **(C)** ELISA-based confirmation of the S2 specificity of 4A5 after final subcloning. **(D)** Detection of SARS-CoV-2 S protein from HEK293T cells using anti-S2 antibody 4A5. SARS-CoV-2 S protein expression was confirmed by probing with anti-His antibody. **(E)** 4A5-detection of cell surface-expressed SARS-CoV-2 S protein (Wuhan or D614G variant) (**Figure S2C**). **(F)** Competitive ELISA examined the presence of 4A5-like antibody in mice sera immunized with SARS-CoV-2 IAV, S2 subunit, or S-trimer. Sera from unimmunized mice of the same age were used as negative controls. ELISAs were performed in duplicates. Data are presented as mean ± SD.

B-cell hybridomas were produced using BALB/c mice vaccinated with β-propiolactone-inactivated SARS-CoV-2 (IAV) (**Figure 1A**). IAV retains the spherical shape of the virus or polymorphic morphology to contain both pre- and postfusion S proteins on the surface (21). Although concerns have been raised about aggregation of virus and loss of antigenic potential that occurs during viral inactivation (20), we expected that the different epitopes contained in inactivated viruses would induce distinct antibodies similar to those generated during viral infection. We screened hybridomas with HEK293T-expressed S2-ECD, selected one hybridoma producing an anti-S2 antibody named 4A5 (**Figure 1B**), and purified this for further study. The isotype was identified as IgG2a.

4A5 specifically detected S2-ECD (686–1213 aa), which resembles the postfusion S protein, as shown via ELISA and western blotting (**Figures 1C**, **S2A**). 4A5 also detected SARS-CoV-2 S (1–1273 aa) protein including ECD and transmembrane (TM) domain expressed in HEK293T cells, which resembles the prefusion conformation of the S protein on the virion, as shown in western blotting (**Figure 1D**) or immunoprecipitation analysis (**Figure S2B**). 4A5 could detect the S protein D614G variant as well as Wuhan type expressed in HEK293T cells (**Figures 1E**, **S2C**).

To verify whether anti-S2 antibodies similar to 4A5 were elicited by other types of SARS-CoV-2 vaccination, we conducted a competitive ELISA for SARS-CoV-2-related antisera using the S2 antigen as the coating antigen and HRP-conjugated 4A5 as the detector antibody (**Figures S2D, E**). Of course, antisera from IAV-immunized mice showed a 4A5-like activity (**Figure 1F**). The 4A5-like antibody activity of 1 µL of IAV-immunized antiserum reached 70% of the standard reaction, which is approximately equivalent to 100 ng of 4A5 antibody (**Figures 1F**, **S2E**). The 4A5-like activity of the antisera against the S2 antigen was also similar to that of IAV-immunized antisera (**Figure 1F**). The S protein trimer, which maintains the prefusion conformational stability of the S protein (22), also elicited a 4A5-like activity, although this was slightly lower than that of S2 antigen, at approximately 40% of the standard reaction (**Figure 1F**). We therefore confirmed that 4A5-like antibodies targeting the S2 subunit were elicited by various vaccinations that included the SARS-CoV-2 S2 subunit as well as IAV. The sequences of complementarity-determining region (CDRs) of heavy and light chain of 4A5 were identified as shown in **Figure S2F**.

### 4A5 showed selective reactivity to S protein of SARS-CoV-2

The epitopes in the S2 subunit are more conserved than those in the S1 subunit (15). Therefore, neutralizing antibodies (nAbs) targeting S2 epitopes would more probably act as broad-spectrum nAbs to SARS-CoV-2 and other β-CoVs. For example, S2P6, which was isolated from a patient recovering from COVID-19, could broadly neutralize β-CoVs, as well as SARS-CoV-2 (9, 17). The S2P6 epitope was identified as 14 residues (1146–1159 aa) in the SH region of the S2 subunit, which is conserved across β-CoVs (17).

**Figure 2:**
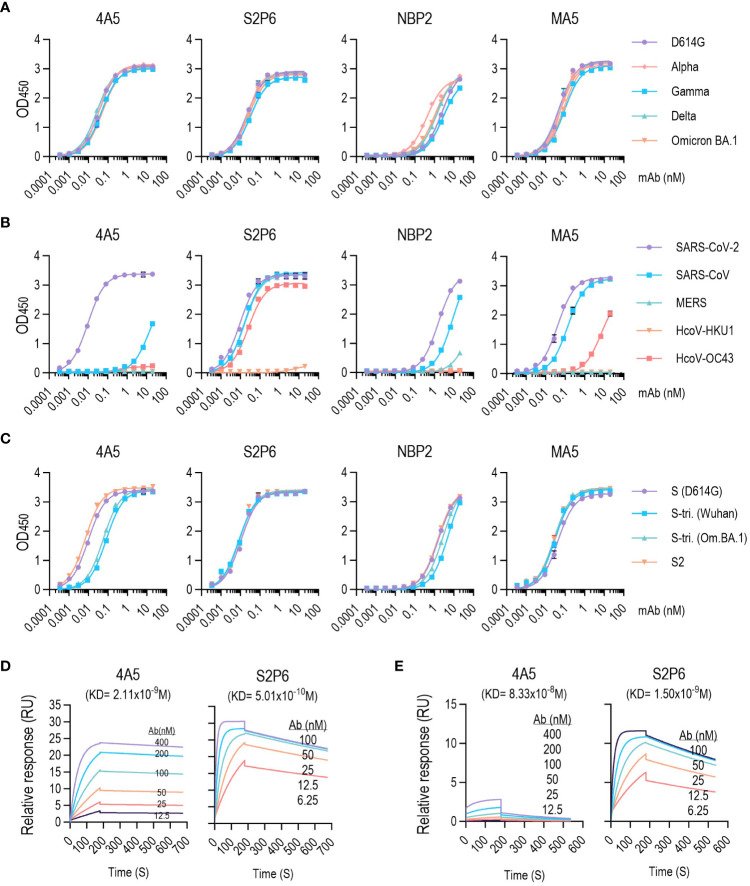
4A5 binds with high affinity to SARS-CoV-2 S variants. **(A–C)** 4A5-binding affinity for different SARS-CoV-2 S protein variants **(A)**, β-CoVs S **(B)** and SARS-CoV-2 trimeric S **(C)** determined via ELISA. EC50 values are listed in **Tables S1–S3**. ELISAs were performed in duplicates. Data are presented as mean ± SD. **(D, E)** 4A5- or S2P6-binding kinetics of immobilized SARS-CoV-2 S **(D)** or SARS-CoV S proteins **(E)** via SPR. KD values were determined using kd/ka values obtained during 180-s association and 500-s dissociation. Kinetic parameters are listed in **Tables S4, S5**.

We compared the binding properties of 4A5 to S variants with other available S2 antibodies, including S2P6. 4A5 binding to the S-ECD (1–1213 aa) of various SARS-CoV-2 variants of concern, including D614G, Alpha, Gamma, Delta and Omicron BA.1, did not differ in ELISA performance (**Figure 2A**, EC50 values shown in **Table S1**). Although the S variants used in the ELISA contained point mutations in the S2 region (S982A and D1118H in Alpha, T1027 and V1176F in Gamma, D950N in Delta and N764K, D796Y, N856K, Q954K, N969K and L981F in Omicron BA.1: Ref. 23) binding of 4A5 to S variants was unaffected. The other S2 antibodies (S2P6, NBP2 and MA5) were also very similar in their binding to the S variants, but the binding of NBP2 to alpha variant was slightly increased.

Unlike the response to SARS-CoV-2 S protein, 4A5 showed no detectable reactivity with other β-CoV S proteins, including from MERS, HCoV-HKU1 or HCoV-OC43 (**Figure 2B**; **Table S2**). 4A5 only detected SARS-CoV S proteins at high concentrations. In contrast, S2P6, which is known to have neutralizing activity against a broad spectrum of β-CoV (17), showed high reactivities to β-CoVs S antigens except HCoV-HKU1. NBP2 or MA5 also exhibited responses to both SARS-CoV and SARS-CoV-2 S proteins but had very low reactivity to MERS or HCoV S proteins.

We also examined the reactivity of 4A5 against prefusion-stabilized S trimers harboring multiple mutations that maintain a highly stable closed conformation of prefusion state S antigen (**Figure 2C**; **Table S3**). Although 4A5 showed a similar activity against the S antigen in the prefusion state or the S2 antigen in the postfusion state conformation, the reactivity against the S trimer (Wuhan or Omicron BA.1) was <5-fold lower, suggesting that the conformational freedom constraints introduced to the S trimer limit the accessibility of the 4A5 antibody. However, other S2 antibodies, notably S2P6, showed similar reactivity to S protein or S trimer.

The SARS-CoV-2 S protein is intrinsically metastable because of it can undergo extensive conformational changes that are required to drive fusion (24). The lower reactivity of 4A5 against the S trimer than against S antigens implies that the antigenic determinant of the 4A5 antibody is affected by conformational changes accompanying the viral infection. To examine the conformational dependency of the 4A5 antigenic epitope, the 4A5 reactivity against denatured S proteins was examined by dot blot analysis or ELISA. Denaturing S antigen with urea or reducing agent significantly reduced 4A5 reactivity. By contrast, the S2P6 reactivity, the epitope of which is mapped to the SH-domain of the S protein, was not significantly affected (**Figures S3A, B**). Thus, the antigenic determinant of 4A5 appears to differ from those of other known S2-antibodies, particularly S2P6.

Antibodies with nonoverlapping epitopes on a target antigen may be suitable for use in an assay aimed at detecting viral antigens or viruses. A sandwich ELISA consisting of HRP-conjugated 4A5 and a capture antibody, S2P6, successfully identified the SARS-CoV-2 S antigen (**Figures S3C, D**). MM43, an antibody specific to the SARS-CoV-2 RBD, was also useful to detect the S antigen when paired with 4A5-HRP. 4A5 could also serve as capture antibody in conjunction with 4A5-HRP for detecting the S antigen because of the trimeric S antigen structure. The same methods were also applicable for detecting SARS-CoV-2 pseudoviruses (**Figures S3C, E**).

4A5-specific binding to the S protein was validated with SPR analysis (**Figures 2D, E**; **Tables S4, S5**). 4A5 and S2P6 exhibited strong binding affinity to SARS-CoV-2 S protein with equilibrium dissociation constants (KD) of 2.11 and 0.5 nM, respectively. The low binding of 4A5 to SARS-CoV S protein was also confirmed (KD = 83.3 nM) and was 40-fold lower than that to SARS-CoV-2 S protein. The affinity of S2P6 to SARS-CoV S protein (KD = 1.5 nM) was similar to its affinity for SARS-CoV-2 S protein.

### 4A5 epitope

**Figure 3:**
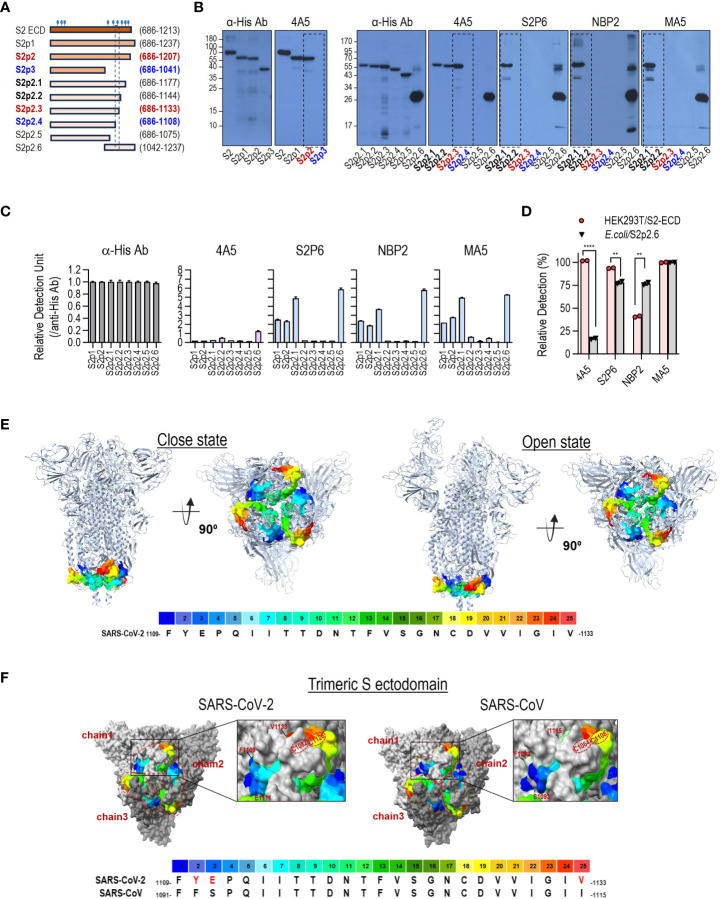
4A5 binds to a conformational epitope in the conserved domain of SARS-CoV-2 S. **(A)** Diagram of the different truncated S2 subunits used to map the epitope recognized by 4A5. The truncated S2 antigens with His-tag were expressed in *E coli.* S2 subunit containing N-glycans expressed in HEK293T cells were used as a control antigen. **(B, C)** Epitope mapping of the 4A5 via western blotting **(B)** or ELISA **(C)** of the truncated S2 subunits. The reactivities of anti-S2 antibodies with characterized epitope sequences were compared to confirm the reported epitopes of these antibodies. In ELISA, the relative reactivity of the antibody to cleaved S2 was corrected by comparing the reactivity of the anti-His antibody to the corresponding antigen. **(D)** The relative reactivity of antibodies to HEK293T-expressed S2 or cleaved S2 (S2p2.6) in ELISA. Relative detection (%) was calculated by comparing the reactivity of the anti-His antibody to the corresponding antigen. ELISAs were performed in duplicates. Data are presented as mean ± SD. *P* values were determined by two-tailed, unpaired t-test and indicated as follows: **** for *P <*0.0001 and ** for P<0.01. **(E)** The 4A5 epitope on the SARS-CoV-2 S structure. The 4A5 epitope (F1109–V1133) is visually represented on the 3D S protein structure using a rainbow color scheme. The prefusion form of the S protein can take on closed or open conformations (PDB: 7DDD and 7DDN, these structures span from Q14 to S1147 of SARS-CoV-2 S protein), with the 4A5 epitope housed at the region underneath the bulb-like head of the S trimer. **(F)** Comparison of structural features of the 4A5 epitope on the trimeric S ectodomains of SARS-CoV-2 S (Q14-S1147; PDB: 7DDD) and SARS-CoV S (T31-L1123; PDB: 8H0X). The 4A5 epitope (F1109–V1133 in SARS-CoV-2 Spike and F1091–I1115 in SARS-CoV spike) was visually depicted on the 3D structure using a rainbow color scheme.

The 4A5 epitope was determined using truncated S2 antigens. Western blotting of truncated S2 proteins showed that 4A5 recognizes the region between 1109 to 1133 (**Figures S1**, **3A, B**). The other three anti-S2 antibodies (S2P6, NBP2 and MA5) recognized an S2 region between amino acids 1145 and 1177 that did not overlap with the 4A5 epitope (**Figure 3B**). These results were confirmed via ELISA (**Figure 3C**). Interestingly, the response of 4A5 to the truncated antigens in the ELISA was relatively lower than that of the other anti-S2 antibodies. Considering that the reactivity of 4A5 to HEK293T-expressed S2 antigen was not significantly different from that of other anti-S2 antibodies (**Figure 3D**), the epitope of 4A5 seems to be involved with conformational features that are not retained in the *E. coli* expression system.

SARS-CoV-2 S variants, including Wuhan, D614G, Gamma, Delta, Omicron BA.1 and Omicron BA.2, share an identical sequence (A1080–T1160) that contains the epitopes of 4A5 and other S2-specific antibodies (**Figure S4A**). The alpha variant has a point mutation (D1118H) in this region, but it did not significantly affect the binding of 4A5 (**Figure 2A**). The corresponding sequences in other β-CoV S proteins differ significantly from those of SARS-CoV-2 (**Figure S4B**). Despite these differences, a consensus sequence is maintained from 1148F to 1156N, which corresponds to S2P6-binding site, a broadly neutralizing epitope for β-CoV (17). Although the 4A5 epitope in SARS-CoV-2 S (F1109–V1133) bears several amino acids identical to those in the S proteins of other β-CoVs, they are located discontinuously (**Figure S4B**), suggesting that they may form different structural features in the spike protein. Therefore, 4A5 binding to S proteins of other β-CoVs including MERS, HcoV-HKU1, and HCoV-OC43, was absent or very low, as shown in **Figure 2B**.

The region corresponding to the 4A5 epitope (F1109–V1133) is located at the bottom of the bulbous head of the prefusion S trimer (**Figure 3E**) and can adopt different conformations in the closed and open states of the prefusion S trimer. The prefusion S trimer structure also contained two closely spaced cysteine residues (C1082 and C1126) that form a disulfide bond (**Figure 3F**). We found that the three terminal amino acid residues of the 4A5 epitope, being different in the SARS-CoV-2 and SARS-CoV S proteins, were closely positioned by this disulfide bond (**Figure 3F**), and that the SARS-CoV-2 and SARS-CoV S proteins retained different structural features from F1109 to V1133. In the postfusion S trimer structure, the 4A5 epitope is located at the head region [connector domain (CD); **Figure S4C**], which may be favorable for the antibody response. The superimposed structure of the postfusion S trimer revealed further distinct structural differences between the 4A5 epitope regions of SARS-CoV-2 and SARS-CoV S proteins, which may explain the differential responses of 4A5 to SARS-CoV-2 and SARS-CoV (**Figure S4D**).

### 4A5 can effectively neutralize S-mediated viral infection and suppress syncytium formation

**Figure 4:**
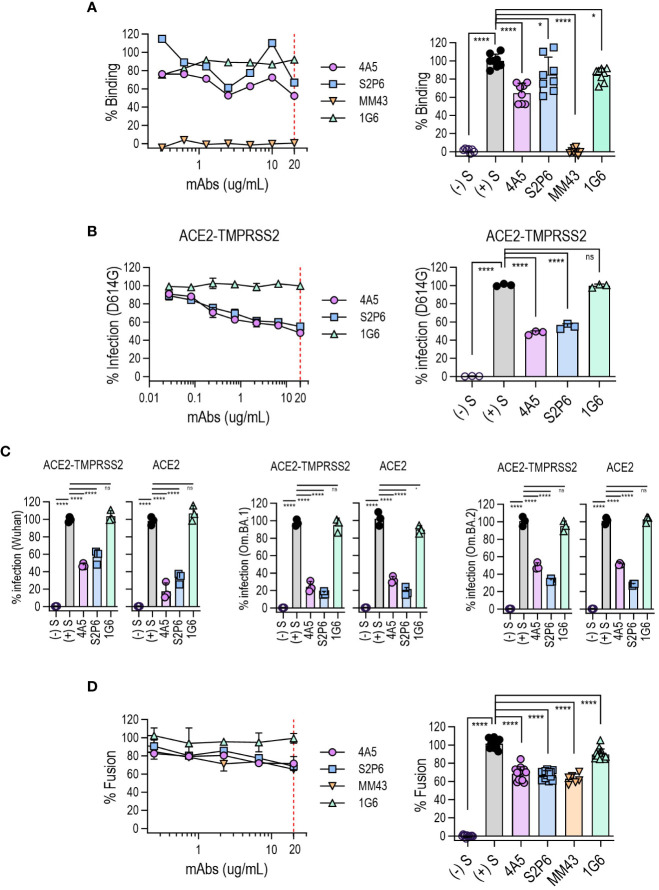
Broad neutralization of SARS-CoV-2 variants by 4A5 and its inhibition of S-mediated membrane fusion. **(A)** SARS-CoV-2 S protein binding to hACE2-overexpressing HEK293T cells was quantified via flow cytometry (% Binding) in the presence of 4A5. Antibody potency was assessed using increasing concentrations of antibodies (left panel), with inhibitory effectiveness compared at 20 µg/mL (n = 8, right panel). Control antibodies S2P6 (anti-S2), MM43 (anti-RBD) and 1G6 (anti-NP) were used. **(B)** 4A5-neutralization of pseudovirus in HEK293T cells expressing hACE2/TMPRSS2. The infection rate of SARS-CoV-2 (D614G) variant was evaluated with increasing antibody concentration (left panel), and the inhibitory potency of antibody was compared at 20 µg/mL (n = 3, right panel). **(C)** Broad neutralizing ability of 4A5 on SARS-CoV-2 variants (Wuhan, Omicron BA.1 and Omicron BA.2). Infection rate was evaluated in hACE2/TMPRSS2 or hACE2-expressing host cells at 20 µg/mL (n = 3; see also **Figures S5A, B** to confirm the pseudovirus titration curve). **(D)** The inhibition of syncytium formation by 4A5 was assessed using 293-hMyD88 cells expressing S protein. Fusion rates were normalized to fusion without mAb and nontransfected cells (left panel) and assessed at 20 µg/mL (n ≥ 6, right panel). In all panels, data are presented as mean ± SD. *P* values were determined by one-way ANOVA multiple comparisons test and indicated as follows: **** for *P <*0.0001, * for *P <*0.1, and ns for *P >*0.9).

To investigate the effect of 4A5 binding to the SARS-CoV-2 S protein during viral infection, we first examined whether the interaction between the S protein and angiotensin-converting enzyme 2 (ACE2) in host cells was influenced by 4A5 binding. Where the binding of the S protein to HEK293T/hACE2 cells was completely blocked by an anti-RBD antibody (MM43), 4A5 inhibited the binding of the S antigen by approximately 40%, whereas S2P6 exhibited approximately 20% inhibition. These results suggest that while 4A5 does not directly bind to the RBD site, its binding affects the structure of the S trimer, subsequently influencing ACE2 binding (**Figure 4A**).

The neutralization potency of 4A5 was also evaluated by whether this inhibited entry of SARS-CoV-2 pseudoviruses into HEK293T/hACE2 in the presence or absence of TMPRSS2 (**Figures 4B, C**, **S5A, B**). 4A5 consistently demonstrated a neutralization effectiveness of 50–80% across all tested variants, including Wuhan, D614G, Omicron BA.1 and Omicron BA.2, regardless of TMPRSS2 presence. Similarly, S2P6 displayed neutralization effectiveness between 50% and 80% across all variants.

The S protein is responsible for mediating the virus entry into host cells by binding to the ACE2 receptor on the cell surface. The S protein can also trigger fusion between infected and uninfected cells, forming multinucleated giant cells termed syncytia (25). Syncytia formation resulting from SARS-CoV-2 infection is a potential contributing factor in the pathogenesis of COVID-19. *In vitro* cell fusion assays revealed that 4A5, S2P6 and anti-RBD antibody MM43 inhibited fusion by approximately 30% (**Figure 4D**). This suggests that anti-S antibodies can contribute to block SARS-CoV-2-induced cell fusion to some extent.

### 4A5 activates Fc-mediated ADCP without ADE

**Figure 5:**
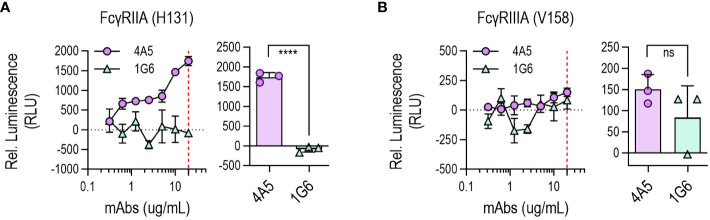
Fc-mediated effector functions of 4A5. Nuclear factor of activated T cells (NFAT)-driven luciferase signals induced in Jurkat cells expressing FcγRIIA [H131; **(A)**] or FcγRIIIA [V158; **(B)**] upon 4A5 binding to SARS-CoV-2 S (D614G) variant expressed in HEK293T cells (n = 3). Data are presented as mean ± SD. *P* values were determined by two-tailed, unpaired t-test and indicated as follows: **** for *P <*0.0001 and ns for *P >*0.9999.

The Fc region of antibodies helps to protect against viruses by promoting immune responses and viral clearance (26). The ability of anti-S2 antibodies to stimulate the Fc receptors, CD16A (FcγRIIIA) or CD32A (FcγRIIA), was estimated by using an *in vitro* assay. S2P6, a human IgG1, triggered ADCC and ADCP to high levels (**Figures S6A, B**) as previously reported (17). 4A5, a mouse IgG2a, also triggered FcγRIIA-mediated ADCP (**Figure 5A**), although FcγRIIIA-mediated ADCC was not induced by 4A5 (**Figure 5B**).

Unlike the typical antiviral defense mechanisms, the Fc-mediated effector function can contribute to ADE by internalizing the virus-antibody complexes into cells through the Fc gamma receptor (FcγR), enabling further virus replication (27). *In vitro* ADE assays were performed using RAW 264.7 or Raji cells, which express FcγRIIA. However, 4A5 as well as S2P6 did not cause FcγR-mediated ADE even at high concentrations (**Figure S6C**).

## Discussion

We have identified a novel anti-S2 antibody, 4A5, which binds to the CD domain of the SARS-CoV-2 S protein, unlike existing anti-S2 antibodies that show specific reactivity to the FP or SH regions. 4A5 binds specifically to the F1109-V1133 sequence in the CD domain and binds effectively to both the postfusion and prefusion states of the S protein. Moreover, 4A5 appears to bind more effectively to SARS-CoV-2 when the virus is ready to bind to host cell receptors, as it is more responsive to the open conformation of the S protein than to the closed conformation of the S-trimer. The epitope sequence of 4A5 is well conserved in most SARS-CoV-2 variants reported to date, allowing it to bind to either SARS-CoV-2 or the S protein regardless of the virus variants. The only mutation identified in the epitope region of 4A5, the D1118H mutation (23), was observed in the alpha strain but did not affect the binding response of 4A5 to S protein. However, its reactivity against other β-CoVs is much lower. This is because the sequence of the 4A5 epitope is highly conserved in SARS-CoV-2 variants, whereas other β-CoVs, including SARS-CoV, have very different sequences in the region corresponding to the 4A5 epitope. On the other hand, the 14 residues in the SH region of the S2 subunit (1146-1159 aa) are well conserved in SARS-CoV-2 and β-CoVs, and anti-SARS-CoV-2 antibodies such as S2P6 that react with this region retain reactivity against β-CoVs.

A number of antibodies against the S2 region have been reported (8, 16), but no antibodies with the same epitope as 4A5 have yet been identified. The S2 antibodies reported so far have been found in convalescent patients with viral infection or in mice immunized with the S antigen. In these cases, antibodies are predominantly produced against the highly immunogenic S1 region, while antibody production against the S2 region is known to be restricted to regions containing SH or FP domains, which induce less potent neutralizing antibodies, possibly due to their inappropriate conformation and/or low immunogenicity (8). We obtained antibodies by immunization with inactivated SARS-CoV-2 viruses. Inactivated viruses retain their S antigen primarily in a postfusion state, so it is likely that elicited antibodies would be directed against epitopes retained in the conformation of postfusion state. Furthermore, as we used the S2 protein for the screening of monoclonal antibodies, it is likely that we were looking for antibodies that would react to the postfusion state conformations. However, the selected antibody, 4A5, also bound well to the prefusion state S protein and inhibited somewhat the binding of the S antigen to ACE2.

These properties make 4A5 suitable for universal diagnosis of SARS-CoV-2 variants regardless of their mutations. In addition, because the 4A5 epitope is present in a trimeric multimeric complex on the virus and the viral S protein, the 4A5 antibody offers the advantage of detecting the virus through a sandwich approach consisting of only 4A5 (**Figures S2D, E**). Of course, it can also be paired with an SH region-specific reactive antibody such as S2P6 to form a diagnostic tool that can differentiate between SARS-CoV-2 and closely related SARS-CoV-1 or β-CoVs.

In addition, the conserved epitope sequence of 4A5 suggests its potential as a broadly neutralizing antibody regardless of SARS-CoV-2 mutations. 4A5 showed 50-80% neutralizing potency against SARS-CoV-2 variants regardless of mutations, which was similar to the potency of the broadly neutralizing antibody S2P6.

4A5 could also inhibit syncytia formation induced by the S protein and could enable Fc-mediated ADCP, thereby validating its effectiveness as a therapeutic antibody. Considering that ADCC and ADCP responses (28) are influenced by Fc receptor binding affinity based on antibody isotype, substituting the isotype of 4A5 from mouse IgG2a to human IgG1 when producing therapeutic antibodies, as with S2P6, could yield antibody therapeutics capable of both ADCP and ADCC. To adapt the 4A5 mouse antibody as a therapeutic antibody, it could be engineered as a humanized antibody or human antibodies targeting the same epitope as 4A5 could be screened.

Given the neutralizing activity of 4A5, a vaccine that elicits 4A5-like antibodies would be expected to contain a broad spectrum of efficacy against SARS-CoV-2 variants. Recently, vaccine strategies targeting the S2 region have received increasing attention to counter SARS-CoV-2, with the SH and FP domains being particularly frequently mentioned (15, 29). In this study, we have shown that epitopes on 4A5 can induce broadly neutralizing antibodies, and we expect that vaccines formulated to induce antibodies targeting this conserved S2 sequence will have improved efficacy.

## Data availability statement

The original contributions presented in the study are included in the article/**Supplementary Material**. Further inquiries can be directed to the corresponding authors.

## Ethics statement

Ethical approval was not required for the studies on humans in accordance with the local legislation and institutional requirements because only commercially available established cell lines were used. The animal study was approved by Institutional Animal Care and Use Committee of KRIBB (approval number: KRIBB-AEC-21003). The study was conducted in accordance with the local legislation and institutional requirements.

## Author contributions

C-KH: Data curation, Formal Analysis, Investigation, Methodology, Writing – original draft, Writing – review & editing. W-HL: Formal Analysis, Investigation, Writing – original draft. JY: Investigation, Resources, Writing – original draft, Writing – review & editing. SS: Investigation, Resources, Writing – original draft. SK: Investigation, Resources, Writing – original draft, Writing – review & editing. D-JK: Funding acquisition, Writing – review & editing. HP: Conceptualization, Funding acquisition, Writing – original draft, Writing – review & editing. E-WC: Conceptualization, Data curation, Formal Analysis, Supervision, Writing – original draft, Writing – review & editing.

## Supplementary material

Figure S1 S2-truncated mutants for 4A5 epitope mapping. DNA fragments obtained by gene amplification of SARS-CoV-2 spike (Wuhan)-His/pCMV3 vector were inserted into pET28a(+) and transformed into T7 Shuffle® *E. coli*. Recombinant protein expression was induced with 1 mM IPTG, and cell lysates in PBS with 1 mM EDTA and 1 mM PMSF were analyzed. The sequences of truncated mutants of SARS-CoV-2 S2 were as follows: S2p1 (686–1237 aa), S2p2 (686–1207 aa), S2p3 (686–1041 aa), S2p2.1 (686–1177 aa), S2p2.2 (686–1144 aa), S2p2.3 (686–1133 aa), S2p2.4 (686–1108 aa), S2p2.5 (686–1075 aa) and S2p2.6 (1042–1237aa).

Figure S2 Characterization of an anti-S2 antibody 4A5. **(A)** Confirmation of S2 specificity of 4A5 antibody. Recombinant viral antigens expressed in HEK293T cells, including SARS-CoV-2 S1, S2, NP and RBD, were analyzed via western blotting with purified 4A5. **(B)** Immunoprecipitation of SARS-CoV-2 S-His with 4A5. HEK293T cell lysates transiently expressing SARS-CoV-2 S-His (1–1273 aa) were immunoprecipitated with 4A5 or an isotype control antibody. Western blot analysis was performed to detect S-His in the immunoprecipitates with rabbit anti-His antibody. Secondary reagent (anti-rabbit IgG-HRP) staining was performed to verify nonspecific reactions before detection of S-His with anti-His antibody. **(C)** Detection of surface-expressed S protein using 4A5 in SARS-CoV-2 S-His-expressing HEK293T cells. Immunofluorescence staining was performed using 4A5 and anti-mouse IgG-Cy5 (left panel), whereas the control group on the right panel was stained only with secondary reagent. **(D)** Competitive ELISA strategy using HRP-conjugated 4A5 to confirm 4A5-like antibody in sera of mice immunized with SARS-CoV-2-related vaccinations. **(E)** Standard curve of the competitive ELISA for quantifying 4A5-like activity. The 4A5-HRP activity was measured against serially diluted 4A5 antibody solutions. ELISAs were performed in duplicates. **(F)** Amino acid sequences of heavy and light chain CDRs (VH on top, VL on bottom) of 4A5. Colored highlights indicate the CDR regions corresponding to CDR H1, H2, H3, L1, L2 and L3.

Figure S3 4A5 epitope structure dependency and detection of SARS-CoV-2. **(A, B)** Reactivity of 4A5 to structural changes in the S protein. 4A5 and S2P6 were incubated with SARS-CoV S-His (SCV-S) and SARS-CoV-2 S-His (SCV2-S), prepared under different conditions: PBS, PBS with 6M urea/2M thiourea or treated with TCEP and iodoacetamide. Antibody reactivity was confirmed via dot blot analysis **(A)** and ELISA **(B)**. BSA was used as a negative control, and the reactivity of the anti-His antibody served as a loading control. The relative affinity of the anti-S2 antibody for spike proteins under denatured conditions was calculated based on the anti-His antibody response to the corresponding antigen containing the His-tag. **(C)** Sandwich ELISA configuration for SARS-CoV-2 S antigen or virus detection using S-reactive capture antibody and 4A5-HRP. **(D)** Sandwich ELISA for SARS-CoV-2 S protein detection using 4A5-HRP. Capture antibodies were coated (100 ng per well) onto a Maxisorp plate, incubated with serially diluted S protein (0.017–1000 ng/mL) and then reacted with 4A5-HRP. The anti-NP antibody 1G6 was employed as a negative control for the capture antibody while anti-RBD antibody MM43 served as a positive control. **(E)** Detection of SARS-CoV-2 pseudovirus variants using sandwich ELISA with 4A5-HRP. Serial dilutions of SARS-CoV-2 pseudovirus (ranging from 6 × 10_6_ to 8 × 10_8_ pseudovirus unit) were used as samples, and the detection rates of S2P6 and MM43 antibodies were calculated in comparison to the 4A5 antibody. In all panels, data are presented as mean ± SD. *P* values were determined by one-way ANOVA multiple comparisons test and indicated as follows: **** for *P* <0.0001, * for *P* <0.1, and ns for *P* >0.9.

Figure S4 Structural features of the 4A5 epitope. **(A, B)** Amino acid sequences for the 4A5 epitope (F1109–V1133) aligned among SARS-CoV-2 variants **(A)** and β -CoVs **(B)**. The epitopes of 4A5 and other S2-specific antibodies are delineated by dashed boxes. Amino acid residues that exhibit consensus in the aligned sequences are highlighted in yellow. Cysteine residues responsible for the formation of the disulfide bond (C1082–C1126) are marked in red. **(C)** Comparison of structural features of the 4A5 epitope on the postfusion conformation of SARS-CoV-2 S (N703-L1197; PDB: 6XRA) and SARS-CoV S (A688-S1178; PDB: 6M3W) protein. The 4A5 epitope (F1109–V1133 in SARS-CoV-2 Spike and F1091–I1115 in SARS-CoV spike) was visually depicted on the 3D structure using a rainbow color scheme. **(D)** Superimposed images of the postfusion state S2 domain of SARS-CoV-2 (PDB: 6XRA) and SARS-CoV (PDB: 6M3W). Amino acid residues within the 4A5 epitope that differ between SARS-CoV-2 and SARS-CoV are marked in red. The distinct sequences are indicated in the superimposed images of postfusion S2 structure using dashed-line box I and II. Residues in SARS-CoV-2 are shaded orange, and those in SARS-CoV are shaded blue.

Figure S5 4A5-neutralising of SARS-CoV-2 pseudovirus. **(A)** 4A5-neutralisation of pseudovirus in host cells expressing ACE2. The infection rate of SARS-CoV-2 D614G variant was evaluated with increasing antibody concentration (left panel), and the inhibitory potency of the antibody was compared at 20 υg/mL (n = 3, right panel). **(B)** Broad neutralizing ability of 4A5 on SARS-CoV-2 variants (Wuhan, Omicron BA.1 and Omicron BA.2). Host cells HEK293T-hACE2 or HEK-Blue_™_ hACE2-TMPRSS2 were used, and infection rates were evaluated through the utilization of serially diluted antibodies (0.027–20 υg/mL) to pseudotyped lentivirus. Anti-S2 antibody S2P6 and anti-NP antibody 1G6 were used as controls. In all panels, data are presented as mean ± SD. *P* values were determined by one-way ANOVA multiple comparisons test and indicated as follows: **** for *P* <0.0001, and ns for *P* >0.9).

Figure S6 Fc-mediated function of S2P6 antibody. **(A, B)** Nuclear factor of activated T cells (NFAT)-driven luciferase signal induced in Jurkat cells expressing FcγRIIIA (V158; **A**) or FcγRIIA (H131; **B**) upon S2P6 binding to SARS-CoV-2 S (D614G) variant expressed in HEK293T cells. **(C)** Functional evaluation of the anti-S2 antibodies (4A5 and S2P6) in antibody-dependent enhancement (ADE). SARS-CoV-2 S (D614G) pseudovirus and serially diluted antibodies (0.027–20 ug/mL) were incubated for 1 h. The mixture was added to RAW 264.7 or Raji cells and cultured for 24 h. ADE activation was evaluated by measuring the luciferase activity generated by the infected pseudovirus. The anti-NP antibody 1G6 was included as a negative control. In all panels, data are presented as mean ± SD.
