# Supplementary figures and images for: Novel S2 subunit-specific antibody with broad neutralizing activity against SARS-CoV-2 variants of concern
Source: Front Immunol. 2023 Dec 8;14:1307693. doi: 10.3389/fimmu.2023.1307693 (PMC10749193; doi:10.3389/fimmu.2023.1307693)

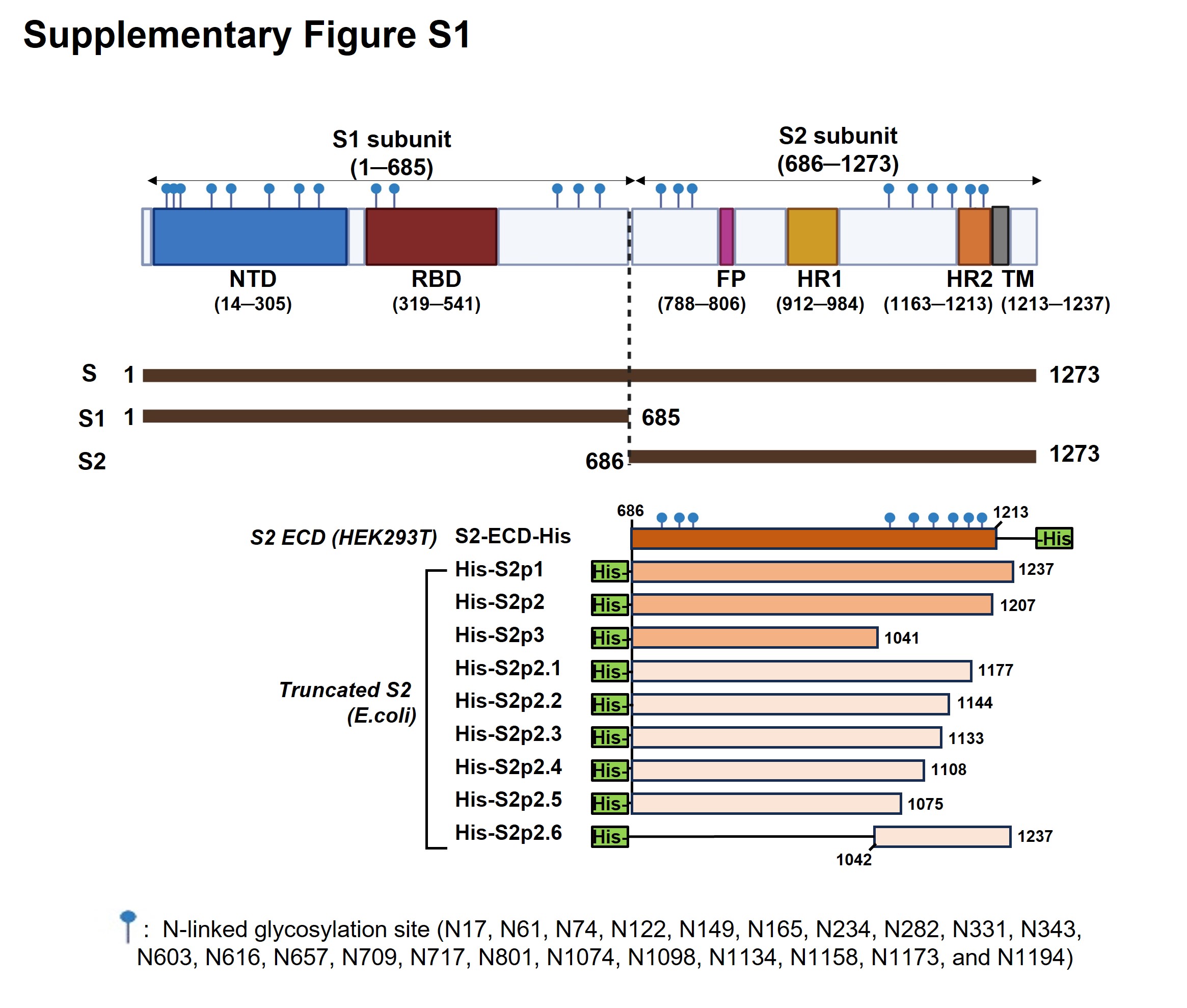

Supplement: Figure S1 — S2-truncated mutants for 4A5 epitope mapping. DNA fragments obtained by gene amplification of SARS-CoV-2 spike (Wuhan)-His/pCMV3 vector were inserted into pET28a(+) and transformed into T7 Shuffle® E. coli. Recombinant protein expression was induced with 1 mM IPTG, and cell lysates in PBS with 1 mM EDTA and 1 mM PMSF were analyzed. The sequences of truncated mutants of SARS-CoV-2 S2 were as follows: S2p1 (686–1237 aa), S2p2 (686–1207 aa), S2p3 (686–1041 aa), S2p2.1 (686–1177 aa), S2p2.2 (686–1144 aa), S2p2.3 (686–1133 aa), S2p2.4 (686–1108 aa), S2p2.5 (686–1075 aa) and S2p2.6 (1042–1237aa). [file Image_1.jpg]

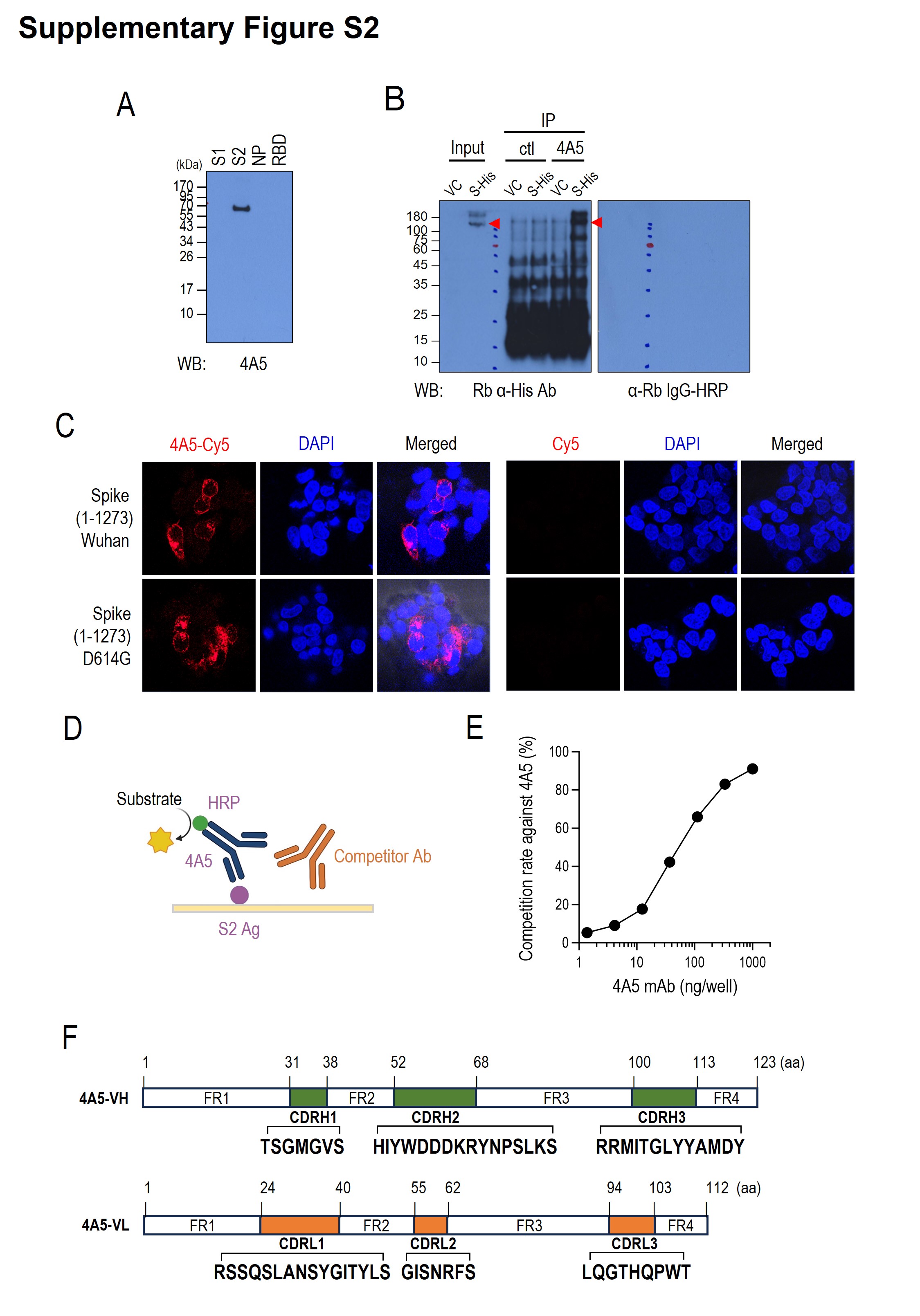

Supplement: Figure S2 — Characterization of an anti-S2 antibody 4A5. (A) Confirmation of S2 specificity of 4A5 antibody. Recombinant viral antigens expressed in HEK293T cells, including SARS-CoV-2 S1, S2, NP and RBD, were analyzed via western blotting with purified 4A5. (B) Immunoprecipitation of SARS-CoV-2 S-His with 4A5. HEK293T cell lysates transiently expressing SARS-CoV-2 S-His (1–1273 aa) were immunoprecipitated with 4A5 or an isotype control antibody. Western blot analysis was performed to detect S-His in the immunoprecipitates with rabbit anti-His antibody. Secondary reagent (anti-rabbit IgG-HRP) staining was performed to verify nonspecific reactions before detection of S-His with anti-His antibody. (C) Detection of surface-expressed S protein using 4A5 in SARS-CoV-2 S-His-expressing HEK293T cells. Immunofluorescence staining was performed using 4A5 and anti-mouse IgG-Cy5 (left panel), whereas the control group on the right panel was stained only with secondary reagent. (D) Competitive ELISA strategy using HRP-conjugated 4A5 to confirm 4A5-like antibody in sera of mice immunized with SARS-CoV-2-related vaccinations. (E) Standard curve of the competitive ELISA for quantifying 4A5-like activity. The 4A5-HRP activity was measured against serially diluted 4A5 antibody solutions. ELISAs were performed in duplicates. (F) Amino acid sequences of heavy and light chain CDRs (VH on top, VL on bottom) of 4A5. Colored highlights indicate the CDR regions corresponding to CDR H1, H2, H3, L1, L2 and L3. [file Image_2.jpg]

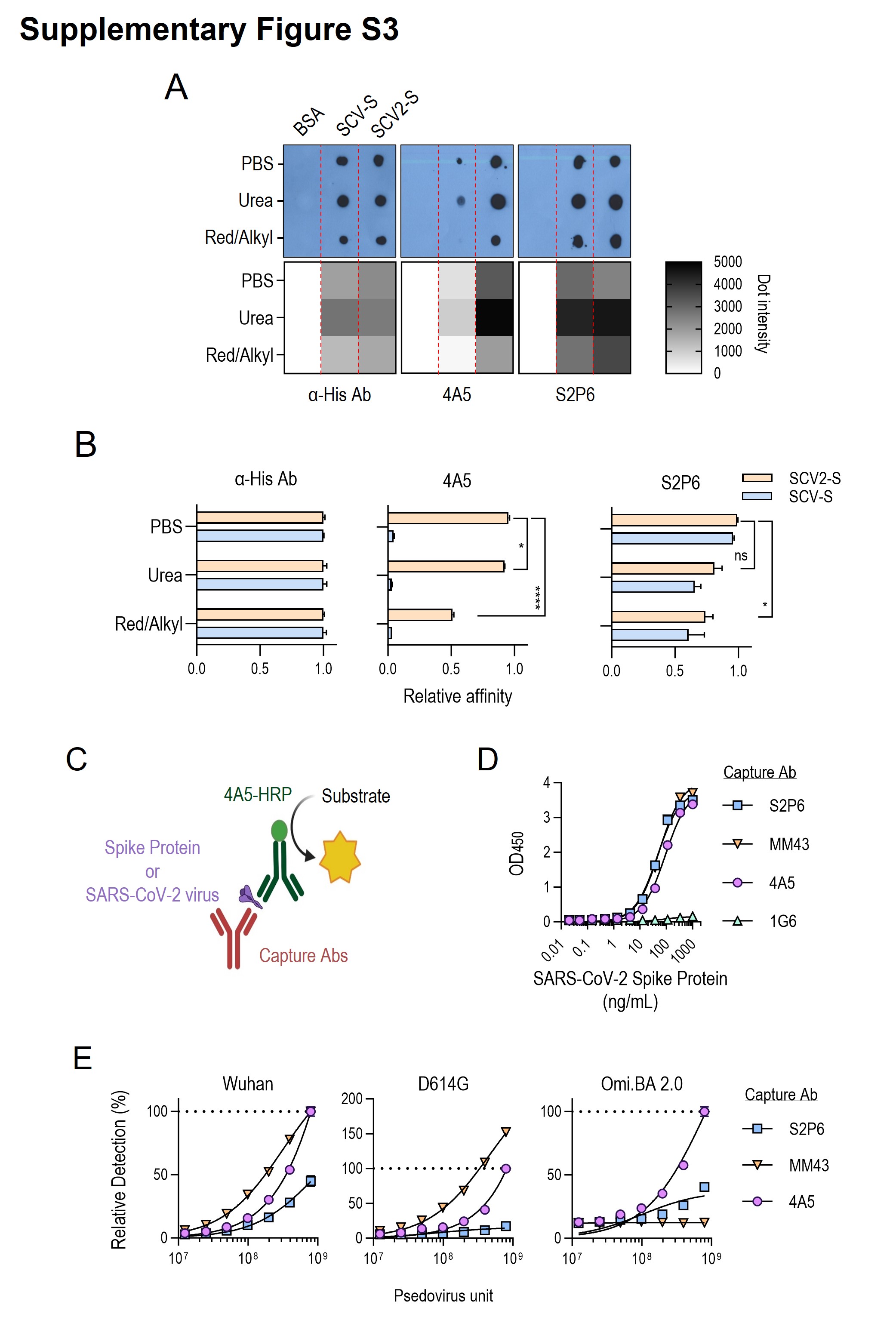

Supplement: Figure S3 — 4A5 epitope structure dependency and detection of SARS-CoV-2. (A, B) Reactivity of 4A5 to structural changes in the S protein. 4A5 and S2P6 were incubated with SARS-CoV S-His (SCV-S) and SARS-CoV-2 S-His (SCV2-S), prepared under different conditions: PBS, PBS with 6M urea/2M thiourea or treated with TCEP and iodoacetamide. Antibody reactivity was confirmed via dot blot analysis (A) and ELISA (B). BSA was used as a negative control, and the reactivity of the anti-His antibody served as a loading control. The relative affinity of the anti-S2 antibody for spike proteins under denatured conditions was calculated based on the anti-His antibody response to the corresponding antigen containing the His-tag. (C) Sandwich ELISA configuration for SARS-CoV-2 S antigen or virus detection using S-reactive capture antibody and 4A5-HRP. (D) Sandwich ELISA for SARS-CoV-2 S protein detection using 4A5-HRP. Capture antibodies were coated (100 ng per well) onto a Maxisorp plate, incubated with serially diluted S protein (0.017–1000 ng/mL) and then reacted with 4A5-HRP. The anti-NP antibody 1G6 was employed as a negative control for the capture antibody while anti-RBD antibody MM43 served as a positive control. (E) Detection of SARS-CoV-2 pseudovirus variants using sandwich ELISA with 4A5-HRP. Serial dilutions of SARS-CoV-2 pseudovirus (ranging from 6 × 10^6^ to 8 × 10^8^ pseudovirus unit) were used as samples, and the detection rates of S2P6 and MM43 antibodies were calculated in comparison to the 4A5 antibody. In all panels, data are presented as mean ± SD. P values were determined by one-way ANOVA multiple comparisons test and indicated as follows: **** for P <0.0001, * for P <0.1, and ns for P >0.9. [file Image_3.jpg]

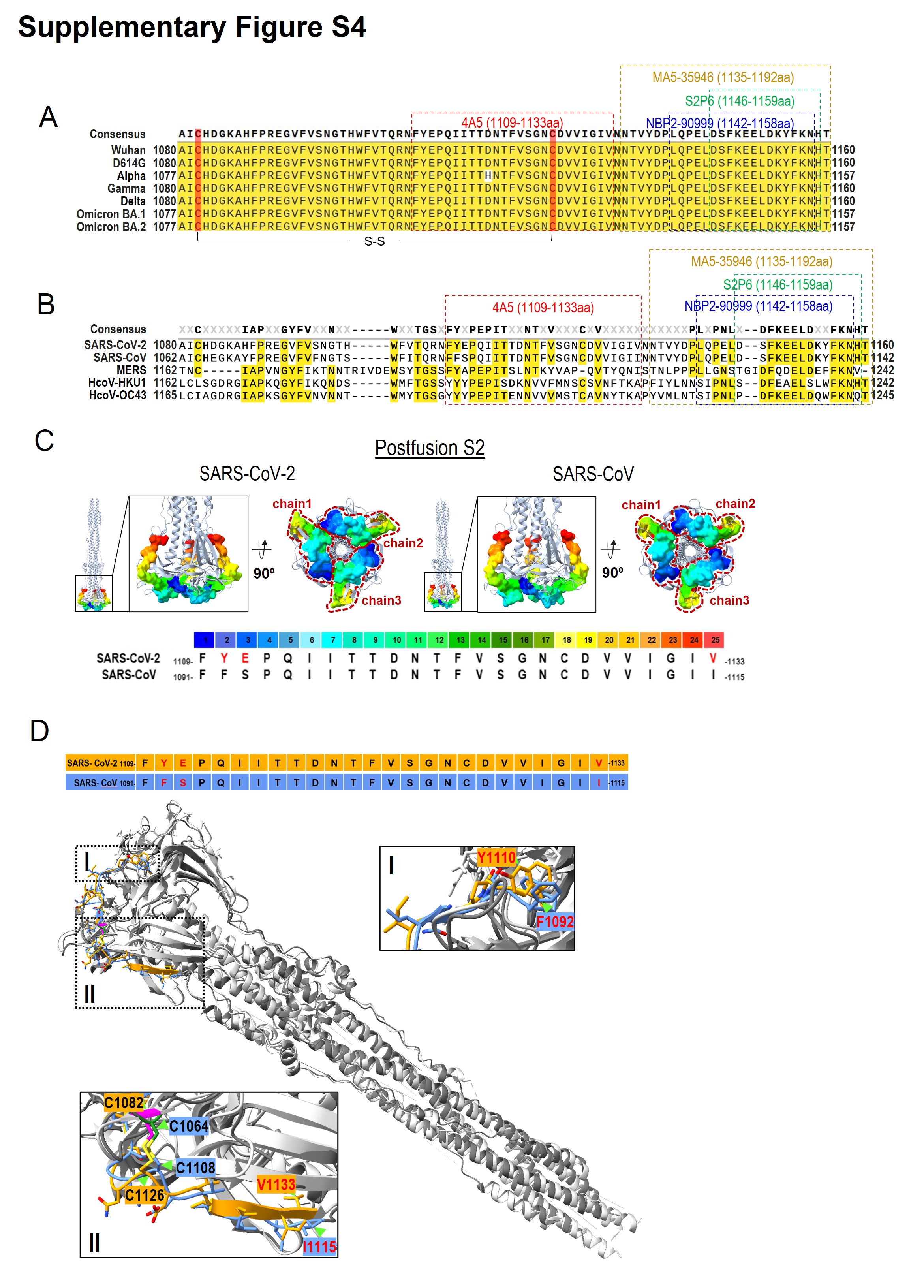

Supplement: Figure S4 — Structural features of the 4A5 epitope. (A, B) Amino acid sequences for the 4A5 epitope (F1109–V1133) aligned among SARS-CoV-2 variants (A) and β -CoVs (B). The epitopes of 4A5 and other S2-specific antibodies are delineated by dashed boxes. Amino acid residues that exhibit consensus in the aligned sequences are highlighted in yellow. Cysteine residues responsible for the formation of the disulfide bond (C1082–C1126) are marked in red. (C) Comparison of structural features of the 4A5 epitope on the postfusion conformation of SARS-CoV-2 S (N703-L1197; PDB: 6XRA) and SARS-CoV S (A688-S1178; PDB: 6M3W) protein. The 4A5 epitope (F1109–V1133 in SARS-CoV-2 Spike and F1091–I1115 in SARS-CoV spike) was visually depicted on the 3D structure using a rainbow color scheme. (D) Superimposed images of the postfusion state S2 domain of SARS-CoV-2 (PDB: 6XRA) and SARS-CoV (PDB: 6M3W). Amino acid residues within the 4A5 epitope that differ between SARS-CoV-2 and SARS-CoV are marked in red. The distinct sequences are indicated in the superimposed images of postfusion S2 structure using dashed-line box I and II. Residues in SARS-CoV-2 are shaded orange, and those in SARS-CoV are shaded blue. [file Image_4.jpg]

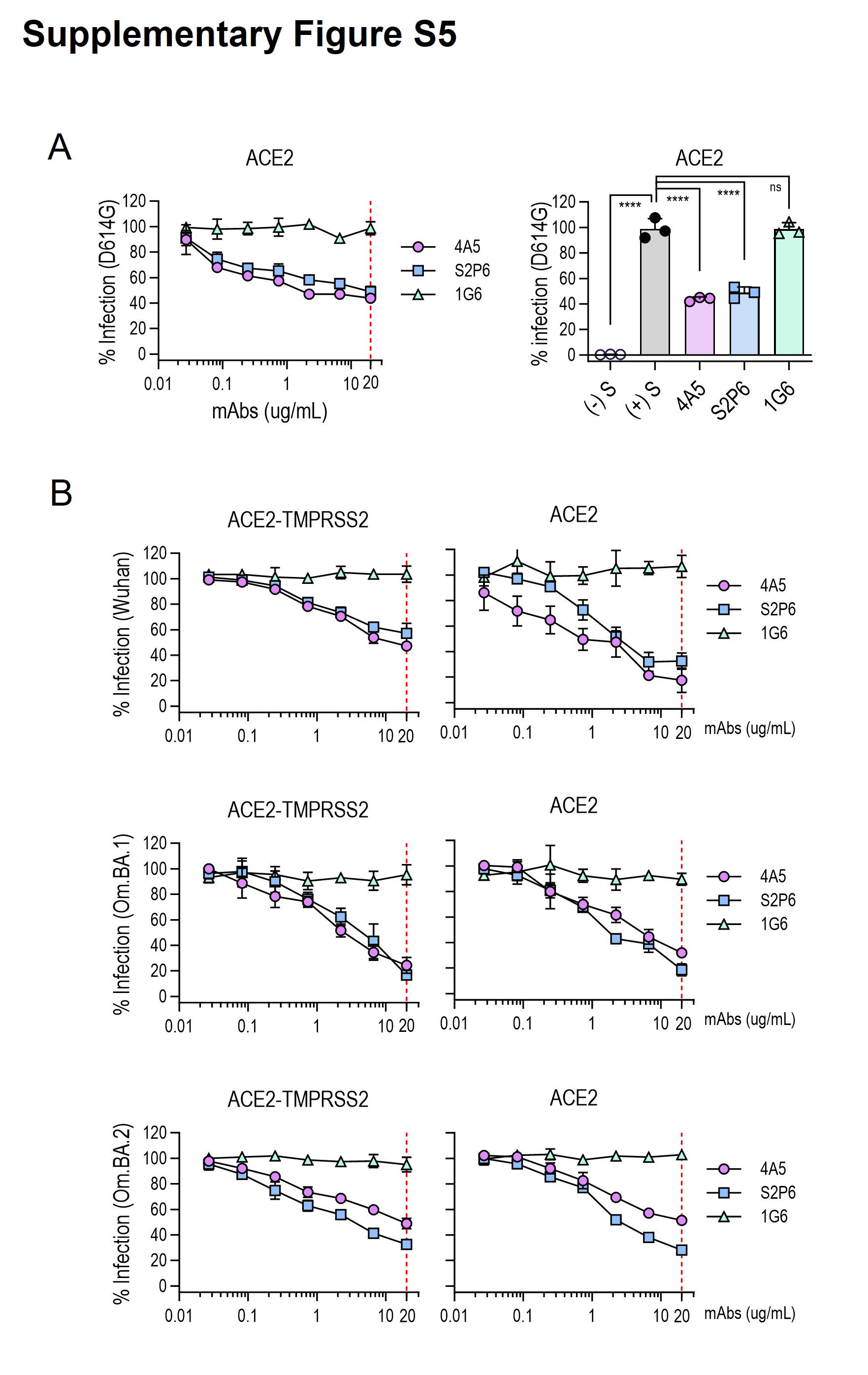

Supplement: Figure S5 — 4A5-neutralising of SARS-CoV-2 pseudovirus. (A) 4A5-neutralisation of pseudovirus in host cells expressing ACE2. The infection rate of SARS-CoV-2 D614G variant was evaluated with increasing antibody concentration (left panel), and the inhibitory potency of the antibody was compared at 20 υg/mL (n = 3, right panel). (B) Broad neutralizing ability of 4A5 on SARS-CoV-2 variants (Wuhan, Omicron BA.1 and Omicron BA.2). Host cells HEK293T-hACE2 or HEK-Blue™ hACE2-TMPRSS2 were used, and infection rates were evaluated through the utilization of serially diluted antibodies (0.027–20 υg/mL) to pseudotyped lentivirus. Anti-S2 antibody S2P6 and anti-NP antibody 1G6 were used as controls. In all panels, data are presented as mean ± SD. P values were determined by one-way ANOVA multiple comparisons test and indicated as follows: **** for P <0.0001, and ns for P >0.9). [file Image_5.jpg]

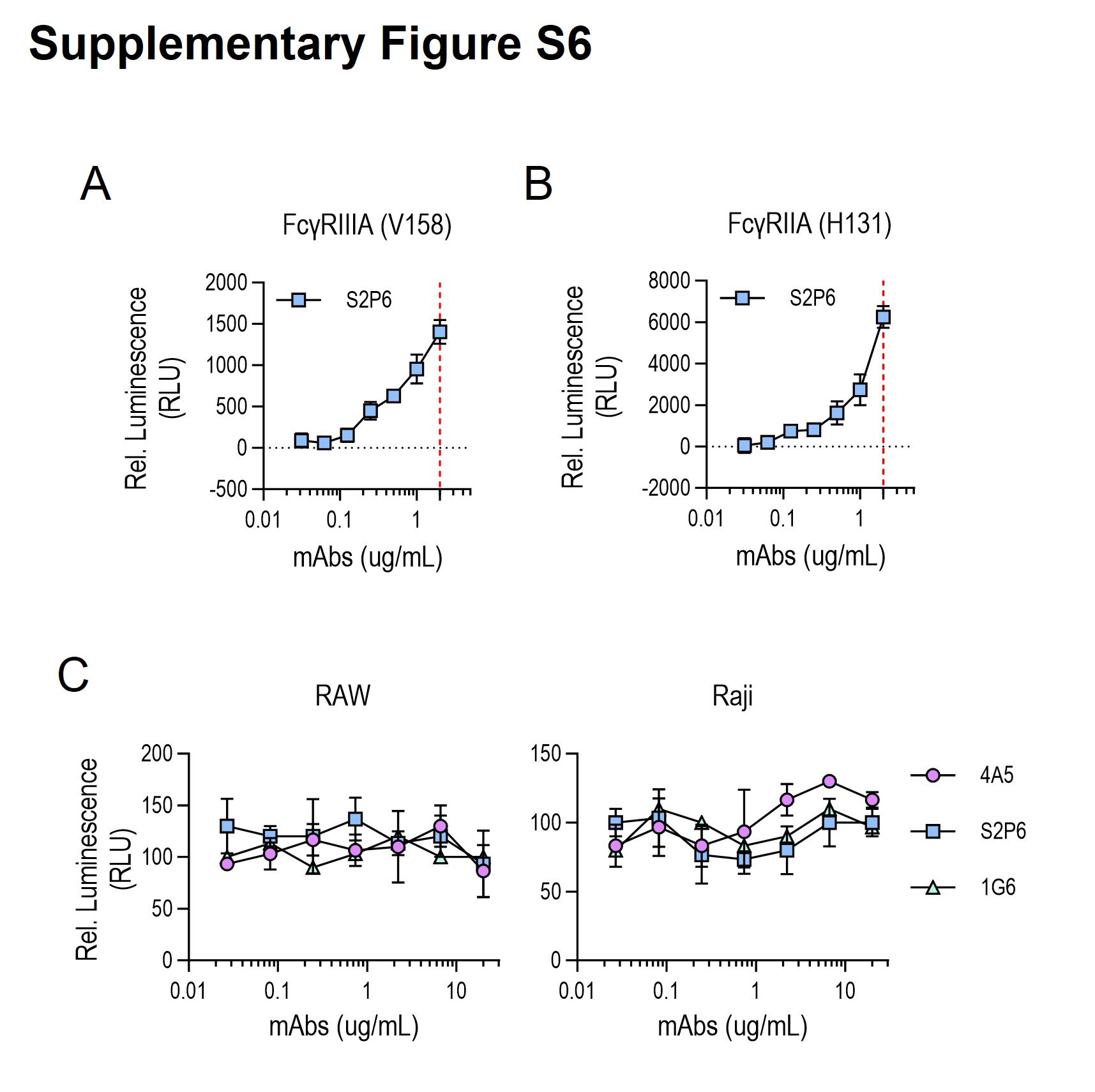

Supplement: Figure S6 — Fc-mediated function of S2P6 antibody. (A, B) Nuclear factor of activated T cells (NFAT)-driven luciferase signal induced in Jurkat cells expressing FcγRIIIA (V158; A) or FcγRIIA (H131; B) upon S2P6 binding to SARS-CoV-2 S (D614G) variant expressed in HEK293T cells. (C) Functional evaluation of the anti-S2 antibodies (4A5 and S2P6) in antibody-dependent enhancement (ADE). SARS-CoV-2 S (D614G) pseudovirus and serially diluted antibodies (0.027–20 ug/mL) were incubated for 1 h. The mixture was added to RAW 264.7 or Raji cells and cultured for 24 h. ADE activation was evaluated by measuring the luciferase activity generated by the infected pseudovirus. The anti-NP antibody 1G6 was included as a negative control. In all panels, data are presented as mean ± SD. [file Image_6.jpg]
