# Supplementary material for: Novel S2 subunit-specific antibody with broad neutralizing activity against SARS-CoV-2 variants of concern
Source: Front Immunol. 2023 Dec 8;14:1307693. doi: 10.3389/fimmu.2023.1307693 (PMC10749193; doi:10.3389/fimmu.2023.1307693)
Supplement: Supplementary file 7 [file Table_1.docx]

**Supplementary Tables**

| **TABLE S1. EC50 (nM) of α-S2 antibodies against SARS-CoV-2 Spike variants.** | | | | | |
| --- | --- | --- | --- | --- | --- |
| **mAb** | **EC50 (nM)** | | | | |
|  | **D614G** | **Alpha** | **Gamma** | **Delta** | **Omicron BA.1** |
| 4A5 | 0.0350 | 0.0358 | 0.0457 | 0.0274 | 0.0530 |
| S2P6 | 0.0219 | 0.0218 | 0.0312 | 0.0211 | 0.0262 |
| NBP2 | 2.4750 | 0.4212 | 2.8570 | 1.4070 | 1.0730 |
| MA5 | 0.0490 | 0.0575 | 0.0882 | 0.0461 | 0.0803 |

| **TABLE S2. EC50 (nM) of α-S2 antibodies against β-CoV Spike proteins.** | | | | | |
| --- | --- | --- | --- | --- | --- |
| **mAb** | **EC50 (nM)** | | | | |
|  | **SARS-CoV-2** | **SARS-CoV** | **MERS** | **HcoV-HKU1** | **HcoV-OC43** |
| 4A5 | 0.0108 | 18.0500 | ND^*^ | ND | ND |
| S2P6 | 0.0099 | 0.0168 | 0.0167 | ND | 0.0248 |
| NBP2 | 1.6770 | 9.6450 | ND | ND | ND |
| MA5 | 0.0426 | 0.1407 | ND | ND | 8.3480 |

^*^ND; Not determined

| **TABLE S3. EC50 (nM) of α-S2 antibodies to SARS-CoV-2 Spike protein.** | | | | |
| --- | --- | --- | --- | --- |
| **mAb** | **EC50 (nM)** | | | |
|  | **S**  **(D614G)** | **S2** | **S-tri.**  **(Wuhan)** | **S-tri.**  **(Omicron**  **BA.1)** |
| 4A5 | 0.0108 | 0.0071 | 0.0827 | 0.0597 |
| S2P6 | 0.0110 | 0.0093 | 0.0091 | 0.0095 |
| NBP2 | 1.6770 | 1.5780 | 5.6540 | 2.8480 |
| MA5 | 0.0426 | 0.0282 | 0.0312 | 0.0291 |

| **TABLE S4. Kinetic parameters of α-S2 antibodies to SARS-CoV-2 Spike protein.** | | | |
| --- | --- | --- | --- |
| **SARS-CoV-2 Spike** | **4A5** | **S2P6** | **comparison** |
| *k*_a_ (1/Ms) | 5.55E+04 | 8.94E+05 | 16.12 times S2P6 > 4A5 |
| ***k*_d_ (1/s)** | 1.17E-04 | 4.48E-04 | **3.83 times 4A5 > S2P6** |
| KD (M) | **2.11E-09** | **5.01E-10** | **4.21 times S2P6 > 4A5** |

| **TABLE S5. Kinetic parameters of α-S2 antibodies to SARS-CoV Spike protein.** | | | |
| --- | --- | --- | --- |
| SARS-CoV Spike | **4A5** | **S2P6** | **comparison** |
| *k*_a_ (1/Ms) | 3.65E+04 | 6.22E+05 | 17.03 times S2P6 > 4A5 |
| ***k*_d_ (1/s)** | 3.04E-03 | 9.32E-04 | **3.26 times S2P6 > 4A5** |
| KD (M) | **8.33E-08** | **1.50E-09** | **55.50 times S2P6 > 4A5** |
